# Synthetic *vs.* natural antimicrobial agents for safer textiles: a comparative review

**DOI:** 10.1039/d4ra04519j

**Published:** 2024-09-26

**Authors:** Aqsa Bibi, Gul Afza, Zoya Afzal, Mujahid Farid, Sajjad Hussain Sumrra, Muhammad Asif Hanif, Bedigama Kankanamge Kolita Kama Jinadasa, Muhammad Zubair

**Affiliations:** a Department of Chemistry, University of Gujrat Pakistan 50700 Pakistan aqsa155991234@gmail.com gulafza80@gmail.com zoyaafzal30@gmail.com sajjadchemist@uog.edu.pk muhammad.zubair@uog.edu.pk; b Department of Environmental Science, University of Gujrat 50700 Pakistan mujahid.farid@uog.edu.pk; c Department of Chemistry, University of Agriculture 38400 Pakistan drmuhammadasifhanif@gmail.com; d Department of Food Science and Technology (DFST), Faculty of Livestock, Fisheries & Nutrition (FLFN), Wayamba University of Sri Lanka Makandura Gonawila Sri Lanka jinadasa76@gmail.com

## Abstract

Textiles in all forms act as carriers in transmitting pathogens and provide a medium of microbial growth, especially in those fabrics which are used in sports, medical and innerwear clothing. More attention towards hygiene and personal healthcare made it a necessity to develop pathogen-free textiles. Synthetic and natural antimicrobial compositions are used to control and reduce microbial activity by killing or inhibiting microbial growth on textiles. Synthetic metallic nanoparticles of Ag, Zn, Cu Ti and Ga are the most commonly and recently used advanced nanocomposites. Synthetic organic materials such as triclosan, quaternary ammonium compounds, polyhexamethylene biguanide, and *N*-halamines have proven antimicrobial activity. Carbon quantum dots are one of the advanced nanomaterials prepared from different kinds of organic carbon material with photoluminescence efficiency also work efficiently in antimicrobial textiles. A greener approach for producing natural antimicrobial textiles has gained significant importance and demand for personal care due to their less toxic effects on health and the environment In comparison to synthetic. The naturally existing materials including extracts and essential oils of plants have significant applications for antimicrobial textiles. Additionally, a number of animal extracts are also used as antimicrobial agents include chitosan, alginate, collagen hydrolysate to prepare naturally treated antimicrobial textiles. This review focuses on the comparative performance of antimicrobial fabrics between synthetic and natural materials. Textiles with synthetic substances cause health and environmental concerns whereas textiles treated with natural compositions are more safe and eco-friendly. Finally, it is concluded that textiles modified with natural antimicrobial compositions may be a better alternative and option as functional textiles.

## Abstract 2

Textiles in all forms act as carriers in transmitting pathogens and provide a medium of microbial growth, especially in those fabrics which are used in sports, medical and innerwear clothing.

## Graphical abstract

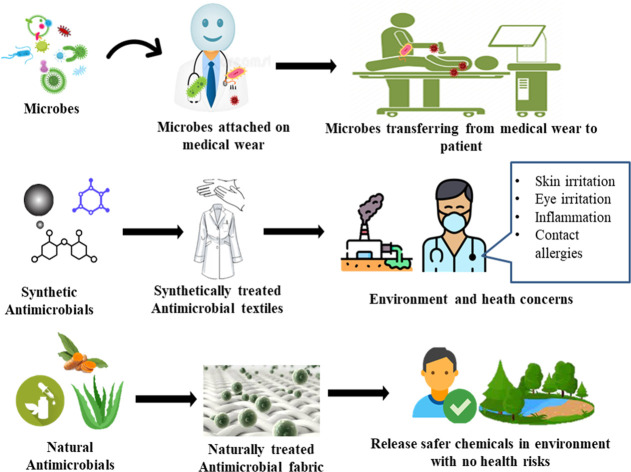

## 1. Introduction to antimicrobial and antiviral textiles

“Functional textiles,” another name for functional fabrics, are materials with integrated components for managing or altering a certain use. Functional fabrics come in enormous varieties these days, including anti-microbial, anti-wrinkle, stain-resistant, flame-retardant, temperature-regulating, and water-repellent varieties. This review focuses on antimicrobial textiles that may be modified by synthetic or natural compositions and compared for safer health and the environment. Textiles don't only perform the function of wearing or styling but they have various other applications in many areas including medical wear, sportswear, military wear and many other fields. These specialized textiles for specific functions are prepared by specialized techniques using agents that are crucial to performing a specific role.^1^ Textile products consist of yarn, fiber and filament which are made from natural and man-made fibrous materials. Textiles that are active against microbes are becoming more and more significant in every business, including the food, automotive, sports, and medical fields, as well as the medical area.^2^ Textiles can be classified as natural and synthetic textiles which are being used in the form of cotton, wool, silk nylon *etc*.^3^ Textile of all types acts as a carrier in transmitting pathogens because it is the most common media for microbial growth especially in those fabrics which are used in sportswear, medical wear and innerwear clothing.^4^

The microbial-contaminated textiles produce various types of infections, cross-infection, and infectious diseases in human bodies such as nosocomial infection. This infection is commonly observed in persons who were treated in a hospital and it is considered to be nosocomial if the infection arises in two days after admitting to the hospital or within a month after discharging the hospital. A variety of fungal, bacterial and viral pathogens are cause of such infections.^5^ In health care units some bacteria like *Staphylococci* are transmitted by the polluted particles of personal protective equipment of nurses, bed clothing and curtain clothing while others like *Pseudomonas* are spread by the moist places in the hospital through contact of one person to another. The risk of virus transmission is also possible by respiratory viruses and blood-borne viruses. These viruses may survive on the surface for few hours or days.^6^ In the early pandemic of SARS-COVID-19, the public was advised to wear face masks by the government and health professionals. However, due to economic reasons and the limited availability of surgical masks, people have to wear handmade woven or knitted cloth masks. These masks have no filtering capacity as these can entrap aerosols of infected persons which was also a reason for the transmission of the corona virus. New antiviral textiles are required for the protection and prevention of life-threatening viral illnesses, as the COVID-19 pandemic demonstrates the necessity of producing enhanced protective equipment.

The demand for antimicrobial textiles is increasing with the increasing pathogenic effect and these textiles play a significant role to limit microbial contact. These infections are of great interest because of textiles use especially in medical health care units, laboratories or clinics. Health care workers are the carriers of spreading bacteria and viruses because of more exposure to a different types of infectious diseases. For this purpose, it is necessary to develop medical textiles which show a wide range of applications inside and outside the body for example in wound dressing, personal care products, hospital clothing, waterproof surgical gown, gloves, footwear, face mask and apparel.^7^ The hygiene and protection of health care workers are possible by wearing these antimicrobial textiles because they are in direct contact with patients of sports injuries, road accidents and infectious diseases.^8^ The demand of these textiles is rapidly increasing due to specific properties of nontoxicity, biocompatibility and bio-absorbability.

Antimicrobial agents are the agents which were used to resist the growth of microbes and most of them are biocides. There are number of inorganic and organic substances used to reduce microbial activity by killing or inhibiting microbial growth on textiles. Numerous types of inorganic nanoparticles are incorporated in the textile industry to enhance antimicrobial fabrication of textile for example, metal and metal oxides such as silver, silver oxide, copper oxide, zinc oxide and titanium oxides are being used to control bacterial attack by generating reactive oxygen species *via* combining to intracellular protein and directly damaging bacterial cell membrane. Moreover, synthetic polymeric based materials are also used to provide microbial free textiles. But there are various side effects associated with the use of chemical based dyes as they can cause allergic reactions, also some photosensitive antimicrobial materials can change their composition upon exposure to sunlight and can convert from useful form to toxic chemical. For these reasons, numerous natural antimicrobials are being researched as alternatives to chemicals to provide antimicrobial properties to textile surfaces.

## 2. Chemistry of antimicrobial textiles

Natural materials and nanomaterials are found to have antimicrobial and antiviral properties due to their stability, low surface area and various other physical and chemical properties. They contain special qualities that make them perfect for a wide range of uses, such as creating antimicrobial and antiviral fabrics. Textiles treated with an agent that prevents the growth of microbes like bacteria, viruses, and fungi are known as antimicrobial textiles. This may aid in halting the spread of illness and infection. Textiles that have been treated with a chemical that kills or prevents the development of viruses are known as antiviral textiles. This can be crucial in stopping the spread of respiratory viruses like COVID-19 and influenza.

Because of the flare-up of the irresistible infections brought about by various pathogenic microorganisms and the improvement of anti-infection opposition the drug organizations and the analysts are looking for new antibacterial specialists. In the current situation, natural plant extracts and nanoscale materials have arisen up as clever antimicrobial specialists inferable from their high surface region to volume proportion and the remarkable substance and actual properties. Nanotechnology is arising as a quickly developing field with its application in science and innovation to fabricate new materials at the nanoscale level. The utilization of nanoparticles is acquiring force in the current 100 years as they groups characterized compound, optical and mechanical properties. The metallic nanoparticles are generally encouraging as they show great antibacterial properties because of their enormous surface region to volume proportion, which is coming up as the retreat and flow interest in the analysts because of the developing microbial opposition against metal particles, anti-infection agents and the advancement of safe strains. Various sorts of nanomaterials like copper, zinc, titanium, magnesium, gold, alginate and silver have come up however silver nanoparticles have ended up being best as it has great antimicrobial adequacy against microscopic organisms, infections and other eukaryotic miniature organic entities.

## 3. Background of antimicrobial chemicals and textiles

Antibacterial activities by synthesizing cotton fabric having photosensitizers such as porphyrin were investigated which show that photosensitized textiles play important role in bio-medical applications. Photosensitized cellulose cotton textiles can be synthesized by grafting cellulose cotton fabric with porphyrin using cyanuric chloride as a binding material. This is an efficient method for avoiding chemical modification of the cellulose fabric. Upon irradiation of visible light antibacterial activities against *S. aureus* and *E. coli* were tested and *S. aureus* showed promising antimicrobial activity (Ringot *et al.*, 2011). In another study antimicrobial activity on cotton fabric using nanoparticles loaded with herbs *Ocimum sanctum* of water extract was determined. The extract of herb was incorporated into the nanoparticle of sodium alginate chitosan and coated on the cotton fabric by using pad dry cure method. The American Association of Textile Chemists and Colorists (AATCC) test was performed which showed the maximum antimicrobial activity on the cotton fiber with the best washing durability. The nanoparticles encapsulated with herbs show the function of biocontrol agents in cotton fabric against bacteria.^9^ Antimicrobial activities on the polyamide fabrics against the *E. coli* and *S. aureus* bacteria were evaluated by synthesizing silver nanoparticles using stabilizing and reducing agents. Ag metal has the most effective antimicrobial activity due to its high toxicity against bacteria. Antimicrobial properties were successfully found against these bacteria.^10^ The antibacterial activity of the dyed fabrics and the natural dye from the leaf of *Melia composita* was tested against a different type of bacteria *Staphylococcus aureus*, *Bacillus cereus*, *Shigella flexneri*, *Staphylococcus epidermidis*, *Escherichia coli*, *Klebsiella pneumonia*, and *Proteus vulgaris*. Cotton, wool and silk (dyed fabric) along with the impregnation of natural dye were evaluated against the test bacteria. Among all the dyed fabrics silk exhibited a maximum decrease in bacterial efficiency. Hence it was concluded that the application of *M. composita* has maximum antibacterial efficacy in sanitized clothing for protective and medical applications.^11^ Majchrzycka and his coworkers (2017) manufactured reusable antimicrobial nonwoven fabric which can be used for longer use in Respiratory Protective Devices (RPDs) for industrial workers. Scanning electron microscopy was used to evaluate the structural studies of the modified fabric. The modified fabric shows the highest antimicrobial activity against bacteria while the minimum is against moulds. This method is highly efficient for filtering nonwoven together with biocidal properties in developing long-term RPDs.^12^ In a study, the antimicrobial cotton textile was manufactured by applying triclosan and sodium pentaborate pentahydrate on the cotton textile with glucagon as an emulsifying agent. This method is helpful in investigating antimicrobial activity against six bacteria as well as some viruses. The treated fabric was examined through critical analysis and results showed that treated fabrics achieved efficient antimicrobial and antiviral properties.^13^ Another method used the reducing properties of viscose to reduce Au^3+^ to AuNPs and oxidize CHO/OH of cellulose to COOH, providing antimicrobial finishing of viscose fibers through direct formation of AuNPs inside fiber macromolecules without the need for any external agents. The fibers that were treated demonstrated strong suppression of several harmful microorganisms, such as fungus and bacteria. The current method for viscose finishing (pigmentation and antimicrobial activity) has several important advantages: it is one-pot, very simple, economical, green, and industrially viable.^14^ The self-cleaning property of the cotton fabrics with the incorporation of zinc oxide nanoparticles (ZnONPs) preferred over another photocatalyst due to best UV blocking property and antibacterial property. The ZnONPs were added photo-catalytically on cotton fabrics using a dip-pad-dry-cure process.^15^ The antibacterial activity will be assessed by using stabilizer as polyvinylpyrrolidone and the irradiation of gamma rays on the silk fiber amended with silver nitrate. The treated antibacterial silk fibers are used in the surgical clothing, bedsheets and gauze *etc.* The presence of silver nanoparticles will maximize the thermal stability of the treated fiber and can be confirmed by scanning electron microscopy and X-ray diffraction techniques. The treated silk fiber shows maximum antibacterial activity against *S. aureus* and *E. coli* bacteria.^16^ With the increase of emerging infectious diseases, there is a need to secure the lives of healthcare workers by developing antibacterial and antiviral personal protective equipment. The personal protective equipment with a biocidal layer shows greater than 99% efficacy against bacteria, viruses or pathogens either suspended in air or liquid form.^17^ Tayel and his other researchers developed antimicrobial skin-protectant textiles with the application of natural biological agents. The extracted agents were incorporated into the cotton textile to evaluate the antimicrobial potentialities against *Staphylococcus aureus* and *Candida albicans* bacteria. This fabrication of cotton textile is beneficial in the production of hygienic gloves, bandages and other skin protectants.^18^ Zhang and Jiang (2018) reported that quaternary ammonium compound QAC-modified fabric shows improved antibacterial and hydrophobic properties against *E. coli*, *S. aureus* and *B. subtilis*. The nanoparticle-coated fiber was found beneficial in restoring hydrophobicity even after dry cleaning.^19^ Sedighi and his colleagues treated polyester fiber with magnetic and electrically conductive materials. It has the advantage of capping the magnetite particles which minimize the generation of reactive oxygen species (ROS). Hence the treated fabric show 99% antibacterial activity against *S. aureus*. The electrically conducting fabric is wearable and use in biomedical, fuel cell and tissue engineering applications because of its nontoxic behavior.^20^ Pal and his research fellows (2018) in this work investigated the antibacterial property on the cotton fabric by the application of zinc oxide nanoparticles. The results exhibited that the modified cotton fabric have maximum antibacterial activity against *Bacillus subtilis* and *Escherichia coli*.^21^ S. Angeloni (2021) made a study about production of antibacterial disposable textile by using the application of essential oil having antimicrobial, antiviral and anti-inflammation properties were encapsulated within the variety of wall materials. Tea Tree Oil^22^ was encapsulated into the wall of Gum Arabian (GA), β-cyclodextrin (β-CD) and poly-vinyl alcohol (PVA) due to their biodegradable properties. The results showed that depending on the wall material TTO/PVA capsulated material show maximum antibacterial activity against different bacteria as compared to other encapsulated materials.^23^ There are several processes by which antimicrobial textiles can be prepared for various purposes. Durably functionalized cellulosic textiles can now be made to be pollutant-removing, air-permeable, hydrophobic, electrically conductive, photoluminescent, self-cleaning, antibacterial, UV-protective, flame retardant, and stain/water resistant without sacrificing comfort. Novel approaches utilizing a range of functionalizing agents derived from metal salts can accomplish these characteristics. To give cellulosic textiles these particular functions, agents such as metal oxides, metal nanoparticles, and metal–organic frameworks that are generated from metal salts are used.^24^ Cellulose-based textiles can be treated by natural agents such as dyes or by synthetic organic agents to improve the biocidal action of these textiles. It is also appropriate to pre-activate cellulosic textiles or use cross-linkers to increase mechanical qualities and durability^25^. *Scutellaria baicalensis* extract was impregnated ultrasonically on the linen fiber due to green and effectual antimicrobial properties. With the help of comparative analysis, it was found that the synergistic effect of linen fiber treated with SBE and baicalin exhibits greater antimicrobial functionality as compared to just baicalin with the minimum dosage of extract.^26^ Szulc and other researchers (2020) first time used beeswax on cellulosic and polyester fabrics to check their antimicrobial activities. Different microbial strains were used but both treated fabrics show the highest biocidal activity against *Aspergillus niger* mold. These modified fabrics have many applications in sportswear activity, prevention of skin infection in health and other clothing *etc.*^27^ The goal of this method is to create a mesoporous composite that will allow essential oils to release under temperature control. Zeolitic imidazole frameworks@microcrystalline cellulose, or ZIF@MCC, is a mesoporous composite that was effectively created by the fast growth of ZIF based on Zn and Co independently within the MCC matrix. The encouraging results showed that the rapid growth of ZIFs within MCC caused the essential oil to be released under temperature control, extending the release period by more than 10 days. The mesoporous composites that have been chosen will be utilized as reusable tablets to regulate the release of volatile essential oils in heated environments.^28^ The sensitivity of fluorescent fabrics to change color when exposed to UV light makes them particularly desirable for use in military, sensing, and camping applications. Although tetrahydroisoquinoline-containing aromatic compounds are renowned for their biological and pharmacological properties, they are not used as fluorescent materials. In order to create luminous cotton fabrics, the current work focuses on the synthesis of derivatives of tetrahydrothienoisoquinoline and their use in textile technology. The synthesized tetrahydrothienoisoquinoline derivatives were successful in producing long-lasting fluorescent textiles, and they may be used for more sophisticated applications such as technical textiles, biosensors and sensors, smart labelling, and anti-counterfeiting measures.^29^ Palladium nanocluster self-implantation is a novel approach currently being demonstrated for the creation of UV protected cotton fabrics with full shielding performance. Palladium precursor was used in two distinct doses to immobilize palladium nanoclusters *in situ* within native and cationized cotton in strongly acidic and basic conditions. Palladium nanocluster implantation resulted in a superior enhancement of the ultraviolet shielding effect. In order to confirm the role of fabric cationization in the creation of extremely durable UV-protective fabrics, the impact of repeated washing cycles on the colorimetric data and the outcomes of ultraviolet protection was also investigated.^30^ There are several reasons to produce these UV-blocking textiles, as UV radiation can cause dangerous effects, from simple tanning to highly malignant skin cancers.^31^ Another strategy was studied that uses *in situ*/thermal polymerization of benzoxazine (BZs) within a cotton matrix to create cotton fabrics that are water-resistant and UV-protective. There was no discernible wetting of the water droplets. After 4 layers of poly-(Ph-*o*-BZ) and poly-(Ph-*p*-BZ) polymers were added to cotton, the UVPF increased dramatically from 1.4 for untreated cotton to 37.9 and 48.2, respectively. Derivatives of poly-benzoxazines may one day be used to make UV- and water-resistant textiles that could be useful for military clothing.^32^ The superior role of silver and palladium metallic particles in acting as a mordant and achieving the dyed cotton fabrics' excellence in colour fastness with additional functions of antimicrobial potentiality and UV-protective action is currently approved through a methodical study that has been demonstrated. Antimicrobial potency against *Candida albicans*, *S. aureus*, and *E. coli* was determined by measuring the inhibition zone, and samples dyed without a mordant and prepared with either silver or palladium precursors were found to have a reduction percent within the permitted range of excellence (93.11–49.51%).^33^ Another work involved the sequential surface modification of silk materials dyed with silver and palladium precursors to provide superior colour fastness, biocidal properties, and UV resistance. RPN dye was used to colour the ready-made textiles. Outstanding UV-blocking efficacy (UPF, 33.1 and UVB blocking percentage, 97.3%) was attained by the palladium-prepared silk dye. For surface-manipulated coloured samples, biocidal performance against *E. coli*, *S. aureus*, and *Candida albicans* was assessed. The reduction percentage was also judged to be in the range of very good to excellent (94.13–98.27%).^34^ In a different work, optically active PAN-nanopolymer was produced by thermally treating polyacrylonitrile (PAN) for autocatalytic cyclization. This polymer can then be used to make nanofibers *via* solution blow spinning. On the other hand, solution blow spinning is recognized as a method for creating nanofibers with high porosity and a big surface area using a small amount of polymer solution. The nanofibers in their manufactured state demonstrated superior microbicide and photoluminescence. For carbon nanofibers made from PAN-nanopolymer (12.5% wt/vol), the estimated microbial reduction percentages against *S. aureus*, *E. coli*, and *Candida albicans* were 61.5%, 71.4%, and 81.9%, respectively. Thus, the produced fluorescent carbon nanofibers may find use in anti-infective treatment.^35^ The presented work involved manipulating the surface of silk fabrics that had been coloured using either palladium or silver as a substitute for mordant. This was done to give the prepared samples both biocidal and UV resistance. The results of the current investigation show that surface-manipulated dyed samples had the best UV resistance, color fastness, and color strength. Outstanding UV-blocking efficacy was attained by the palladium-prepared silk dye. For surface-manipulated coloured samples, biocidal performance against *E. coli*, *S. aureus*, and *Candida albicans* was assessed. The reduction percentage was also judged to be in the range of very good to excellent (94.13–98.27%).^34^

## 4. Classification of antimicrobial agents used on textiles

**Fig. 1:**
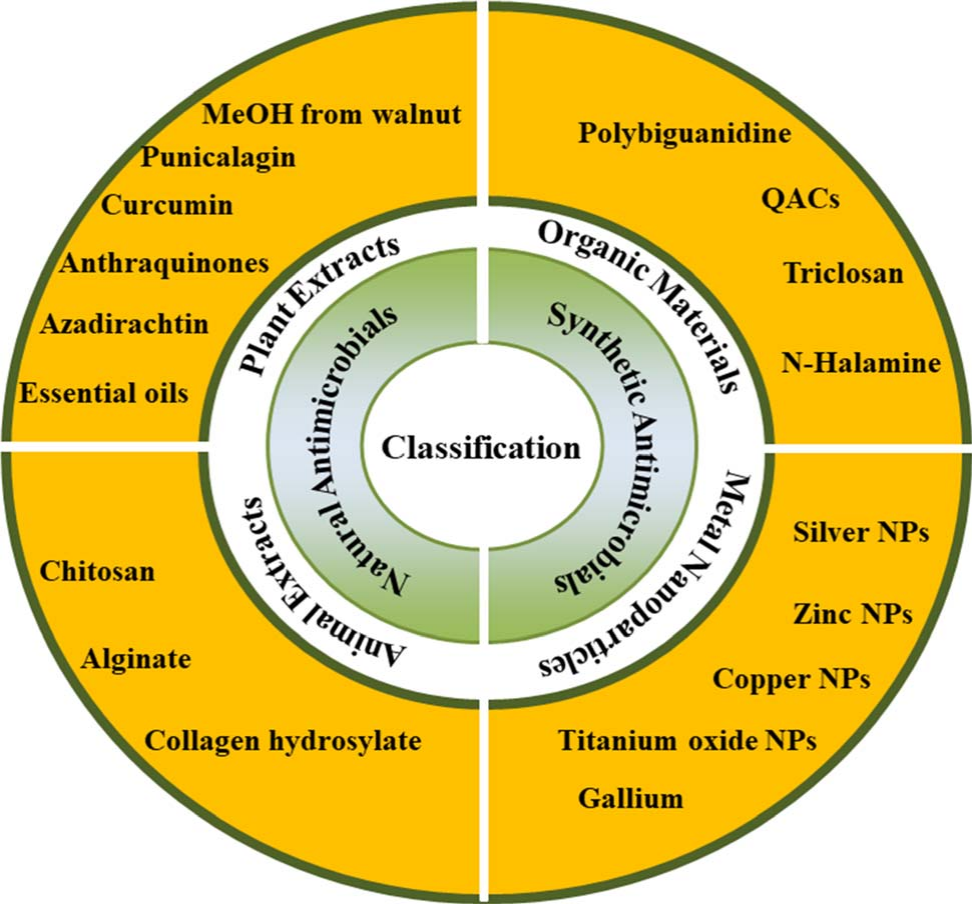
Classification of antimicrobial and antiviral agents.

Numerous antimicrobial substances have been discovered to inactivate viruses in various ways. Antiviral materials are not yet categorized in a standardized manner. According to their chemical makeup, the materials widely utilized as antimicrobial and antiviral agents are separated into synthetic and natural agents as shown in Fig. 1. Synthetic antimicrobial compounds can have a long-lasting impact on textiles and are effective against a variety of microorganisms. They do, however, have drawbacks, such as side effects that may be present, an impact on bacteria that aren't the target, and water contamination. Therefore natural antimicrobial agents are in great demand. Natural antimicrobial agents are better for the environment and human health than synthetic antimicrobial agents. They are less prone to have negative effects like allergic reactions or skin rashes. Additionally, they don't contaminate waterways or help to spread antibiotic resistance. Several applications exist for natural antibacterial agents. They can be added to textiles as a finishing treatment or applied to fabrics during the production process. Compared to synthetic antimicrobial agents, natural antimicrobial agents have a number of benefits. They are more efficient, effective, and safe. They are consequently rising in the textile sector.

## 5. Synthetic materials for antimicrobial textiles

There are a variety of antimicrobial agents used in the textile sector. Antimicrobial agents that are synthesized in the laboratory through various synthetic routes by using chemicals are named as synthetic materials for antimicrobial agents. These antimicrobial agents are in wide spread use because of their various properties such as fast, effective and efficient microbial action, easy preparation because of known procedures, easy approach and long-term use without their deterioration. The most commonly used antimicrobial agents in textile applications are based on metal salts (silver, zinc, copper, titanium oxide, gallium), quaternary ammonium compounds (QAC), polybiguanide (such as PHMB), halogenated phenols (such as triclosan), and *N*-halamines.

### 5.1. Inorganic nanomaterials as antimicrobial and antiviral agents

Inorganic nanomaterials show versatile range of properties that make them capable of combating Antiviral and antimicrobial properties. Inorganic nanomaterials are divided into two categories, metal ions and carbon based nanomaterials. Metal ions incorporated textiles show significant antiviral activity as compared to the carbon based nanomaterials.

#### 5.1.1. Metal based antimicrobial materials

**Fig. 2:**
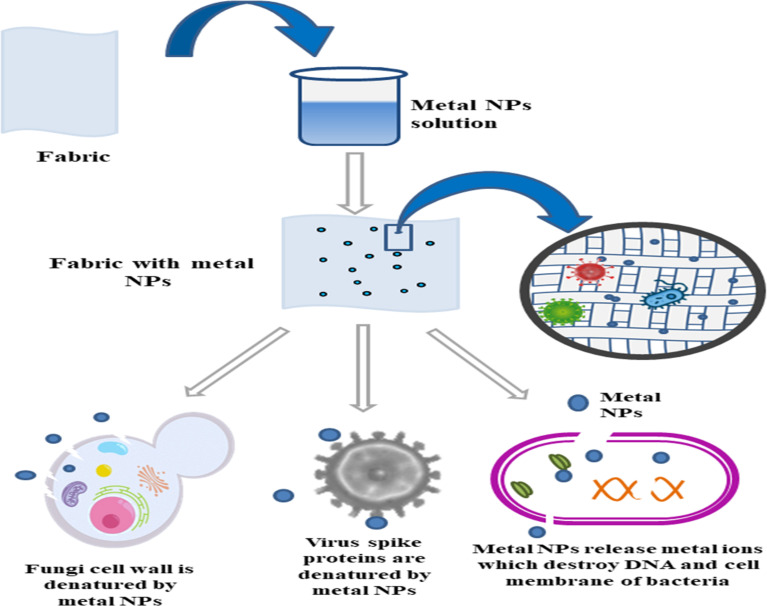
Mechanism of action of metal nanoparticles as antimicrobial agents. Metal nanoparticles inactivate the virus by destroying the spikes and kill the bacteria and fungi by rupturing the cellular structures.

Inorganic metals due to their tiny size and large specific surface area, metal-based have been shown to have special physicochemical properties that enable them to interact with viruses and other microbes. Ag, Zn, Cu, Ti, Ga, are few of the metal and metal oxide nanoparticles that have been used as antiviral agents because to their wide spectrum of antiviral activity, durability, and efficiency at much lower concentrations. They are also helpful in preventing viral infections. Metal based NPs show a significant antiviral activity as compared to the nonmetallic nanoparticles. The mechanism of action of metallic NPs is shown in Fig. 2.

Metal based NPs act by either damaging the cell from the inside or from the exterior site by damaging the protein capsule of virus or bacteriophage. Metal NPs can bind with the virus spike proteins, thus restricting its attachment with the host cell. Also they can penetrate the cell wall of bacteria and release metal ions inside the cell which damages the DNA containing viral genome and also stops protein synthesis by interrupting the ribosomal structure thus preventing the replication of bacteria.^54^ Metal nanoparticles can damage the mannoproteins in cell wall of fungi and can penetrate the cell by endocytosis thus damaging the intracellular structures. Metal nanoparticles when reach inside the cell can release reactive oxygen species which can trigger irreversible damage to cell structures.^55^

##### 5.1.1.1 Silver nanoparticles

For quite a long time silver has been in need for the therapy of persistent injuries. In 1881, Carl S. F. Crede relieved ophthalmia neonatorum utilizing silver nitrate eye drops. Crede's child, B. Crede planned silver impregnated dressings for skin grafting.^56^ In the 1940s, after penicillin was presented the utilization of silver for the treatment of bacterial diseases limited. Silver again came in picture during the 1960s when Moyer presented the utilization of 0.5% silver nitrate for the treatment of consumes. He recommended that this arrangement doesn't disrupt epidermal multiplication and have antibacterial property against *Staphylococcus aureus*, *Pseudomonas aeruginosa* and *E. coli*.^57^ In 1968, silver nitrate was joined with sulfonamide to shape silver sulfadiazine cream, which filled in as an expansive range antibacterial specialist and was utilized for the treatment of consumes. Silver sulfadiazine is compelling against microbes like *E. coli*, *S. aureus*, *Klebsiella* sp., *Pseudomonas* sp. It likewise has a few antifungal and antiviral effects.^58^ As of late, because of the rise of anti-infection safe microorganisms and impediments of the utilization of anti-infection agents the clinicians have gotten back to silver injury dressings containing changing degree of silver Table 1.

**Table 1:** Different synthetic antimicrobial materials used for textile applications^a^

Antimicrobial agents	Textile material	Application	Active against	Ref.
**Chemicals**				
Ag-NPs	Wound dressing, PPE	Corona virus, *S. aureus, E. coli* and *C. albicans*	Bacteria and virus	36–38
Zn-NPs	Facemask	SARS-CoV-2 and influenza A virus	Virus	39 and 40
Cu-NPs	Masks, PPE, socks, wound dressings	Corona virus, *S. aureus, E. coli, L. monocytogenes, S. epidermis,* fungi	Bacteria, virus and fungi	41–43
TiO_2_-NPs	Cotton fabric, PPE, polyester fabrics	*S. aureus*, *E. coli*, methicillin-resistant *Staphylococcus aureus* (MRSA), and *Micrococcus luteus*	Bacteria	44 and 45
Ga-NPs	Masks, gowns, PPE	*S. aureus*, *Escherichia coli*, and *Candida albicans*	Bacteria and fungi	46
Triclosan	Cotton fabrics	Corona virus, bacterial control	Bacteria and virus	47 and 48
Polyhexamethylene biguanidine	Wound dressing	Antiseptic	Bacteria and fungi	49 and 50
QACs	Masks, gloves,PPE	Coronavirus, bacteria	Bacteria and virus	51 and 52
*N*-Halamine	Nonwoven fabric	Avian influenza	Virus	52 and 53

a PPE: personal protective equipment, AgNPs: silver nanoparticles, Cu-NPs: copper nanoparticles, ZnNPs: zinc nanoparticles, QACs: quaternary ammonium compounds.

Silver ties to the bacterial cell wall and cell layer and represses the breath interaction. The silver nanoparticles show proficient antimicrobial property contrasted with different salts because of their very enormous surface region, which gives better contact microorganisms. The nanoparticles get appended to the cell film and furthermore infiltrate inside the microbes. The bacterial layer contains sulfur-containing proteins and the silver nanoparticles connect with these proteins in the cell as well as with the phosphorus containing intensifies like DNA. At the point when silver nanoparticles enter the bacterial cell it frames a low sub-atomic weight district in the focal point of the microorganisms to which the microscopic organisms combinations consequently, safeguarding the DNA from the silver particles.^59^ The nanoparticles ideally assault the respiratory chain, cell division at last prompting cell demise. The nanoparticles discharge silver particles in the bacterial cells, which improve their bactericidal action.

##### 5.1.1.2 Zinc nanoparticles

A person infected with Influenza A viruses and SARS-CoV-2 can spread the disease through liquid droplets and aerosols. Face masks and other protective equipment can help prevent the spread of these viruses, but they can also become contaminated with viruses and need to be disposed off properly. Metal ions embedded in face masks and protective equipment may inactivate respiratory viruses. Polyamide fibers containing embedded zinc ions could inactivate SARS-CoV-2 and Influenza virus by combining with the virus surface protein and thus damages the structure. The zinc content and the virus inactivating property of the fabric remains stable over 50 standardized washes.^39^ Thus zinc-embedded polyamide fabrics could be a promising new material for reusable protective equipment that offers protection against virus spread.

Face masks can become a threat for spreading the Covid-19 as the respiratory droplets of an infected person can cross the respiratory valve in masks and may spread to the nearby environment. This disease spread can be controlled by using zinc oxide nanoparticles in the respiratory valves as a membrane to filter and inactivate the viruses from the exhaled air of the infected person. Zinc oxide in the form of nanoflowers can trap the virus and denature the spike proteins thus causing inactivation of Covid-19 virus.^60^ This nanosynthesized membrane filter does not causes any breathing discomfort along with effective spread control of Covid-19 Table 1.

##### 5.1.1.3 Copper nanoparticles

Ancient cultures employed copper for antibacterial and antiviral purposes. With advancements in textile technology, coating the textile with copper is now a successful approach for metalizing the material and realizing the antibacterial characteristic. COVID-19 is now spreading rapidly over the world. Copper has been proved for certain bacteria and viruses which have significant effect according to several researches, including Human-coronavirus 229E.^41^

Human coronavirus 229E, which causes a variety of respiratory symptoms from the common cold to more fatal outcomes like pneumonia, can survive on surface materials like ceramic tiles, glass, rubber, and stainless steel for at least five days, according to a recently published paper in mBio, a journal of the American Society for Microbiology. Even if hand-to-hand contact or surfaces contaminated by respiratory droplets from sick people can transmit diseases more quickly and widely than human-to-human transmission. The coronavirus was quickly rendered inactive on copper and a variety of copper alloys, which are generally referred to as “antimicrobial copper”. The researchers came to the conclusion that antimicrobial copper surfaces might be used in public spaces and at any large gatherings to help prevent the spread of respiratory viruses and safeguard public health since exposure to copper totally and irrevocably killed the virus. Metal oxide nanoparticles, like copper oxide,^26^ stand out for the most part in view of their antimicrobial and biocide properties and they might be utilized in numerous biomedical applications.

The primary processes behind copper oxides' antibacterial action are still poorly understood. The antibacterial effects of copper oxide are due to a number of mechanisms, including the release of copper ions, direct contact between bacteria and copper oxide, and reactive oxygen species,^61^ which, when in contact with the bacterial cell wall and cytoplasmic membrane, result in cell death. The release of copper ions and the production of ROS may be related to copper's antiviral action. The viral genome can be destroyed, the viral envelopes can fall apart, and copper can stop the multiplication of viruses all of which can result in permanent damage to the viral membrane. Due to the release of the Cu ion, which may harm viral lipid membranes and nucleic acids and render the virus inactive, and the generation of reactive oxygen species (ROS), which are capable of harming viral proteins and lipids, copper has antiviral capabilities.

##### 5.1.1.4 Titanium oxide nanoparticles

Titanium oxide is an antimicrobial agent that is used in cosmetics, pharmaceuticals and several healthcare products. Titanium has many oxides but the most common is TiO_2_ which is most commonly used in antimicrobial textiles. The main advantage of using TiO_2_ is low cost and less toxicity. Gedanken *et al.* investigated the creation of a well-adhered bactericidal TiO_2_ surface on organic cellulose fibers, reporting the antibacterial efficacy of apatite-coated TiO_2_ fixed onto cotton textiles by a dip-coating approach. They suggested that an apatite-coated TiO_2_ suspension's photocatalytic activity might aid in microbial breakdown in textile applications.^62^ When TiO_2_ NPs are placed on the surface of cotton fibers as a coating, which are made up of aggregated nanoparticles with an average size dimension of less than 50 nm. TiO_2_ NPs-loaded cotton textiles have a bacterial reduction of more than 95%, which is sustained even after 20 washing cycles; the bacterial reduction rises when the urea nitrate concentration employed in TiO_2_ NPs production is increased.^63^

##### 5.1.1.5 Gallium nanoparticles

Gallium has drawn significant attention towards textile sector due to its amazing qualities, which include minimal toxicity and great antibacterial efficiency. These characteristics make gallium very appealing for a range of applications, including the medical field. Liquid gallium is coated on fabrics which adheres strongly to the fabric and thus kills 99% of pathogens including bacteria, viruses and fungi within a very short time. The coating of gallium particles on textile surfaces provide nucleation sites to adhere copper ions by galvanic replacement, this dual coating provides far better antimicrobial activity as compared to the single nanomaterial. This antibacterial. Antiviral and antifungal coating remains active over multiple washing cycles.^64^ Ga functions as a synergistic antibacterial agent and as a glue between the cloth and the copper. This study is particularly significant because the antibacterial fabric may be used to make masks, which at this point in time, when COVID-19 is sweeping the world, can significantly minimize viral infection. A bioceramic-liquid metal composite was created by adding gallium as a liquid metal to hydroxyapatite. On textile coatings the mechanical qualities remained unchanged, the gallium species added Ga as an antibacterial agent to the coating. The HAp-Ga coating demonstrated exceptional antibacterial efficacy against both Gram-positive and Gram-negative bacteria.^65^ Another combination that is very effective antimicrobial utilizing gallium is Ag–Ga amalgamated nanoparticles in a suspension that may be sprayed over a range of surfaces to impart antibacterial characteristics. The results showed enhanced efficiency by the combined effect of both nano-materials.^66^ Ga is moving quickly in the direction of broad-spectrum antibiotics to fight viruses, fungus, bacteria, and other microbes.^46^ Gallium–chitosan is an example of such nanocomposites. The antibacterial effectiveness of the chitosan–gallium nanocomposite is reported better than that of controls like chitosan and gallium nitrate. The chitosan–gallium nanocomposite potentially a strong antibacterial agent against *Pseudomonas aeruginosa* infections.^67^

### 5.2. Organic materials as antimicrobial agents

Organic antiviral agents offer the advantages of rapid sterilization, simple production, and great effectiveness. They are often utilized in the synthesis of antiviral medications and in the preparation of antiviral fabrics during textile finishing. Organic substances can be directly converted into antiviral fibers or polymerized and copolymerized with other comonomers to create antiviral coatings. Organic bases anti-microbial agents such as *N*,*N*-dodecyl, methyl-polyurethane when incorporated on surfaces or electrospun into fiber, were able to inhibit the growth of airborne Gram-positive *Staphylococcus aureus* and Gram-negative *Escherichia coli* bacteria, they may also play role in inactivating the Influenza virus. *N*-Halamines was another agent when coated on nonwoven fabrics, it can completely inactivate Avian Influenza^68^ viruses by disrupted their RNA, and were considered to be efficient against the production of airborne pathogens in the poultry environment.^69^

#### 5.2.1. Triclosan

**Fig. 3:**
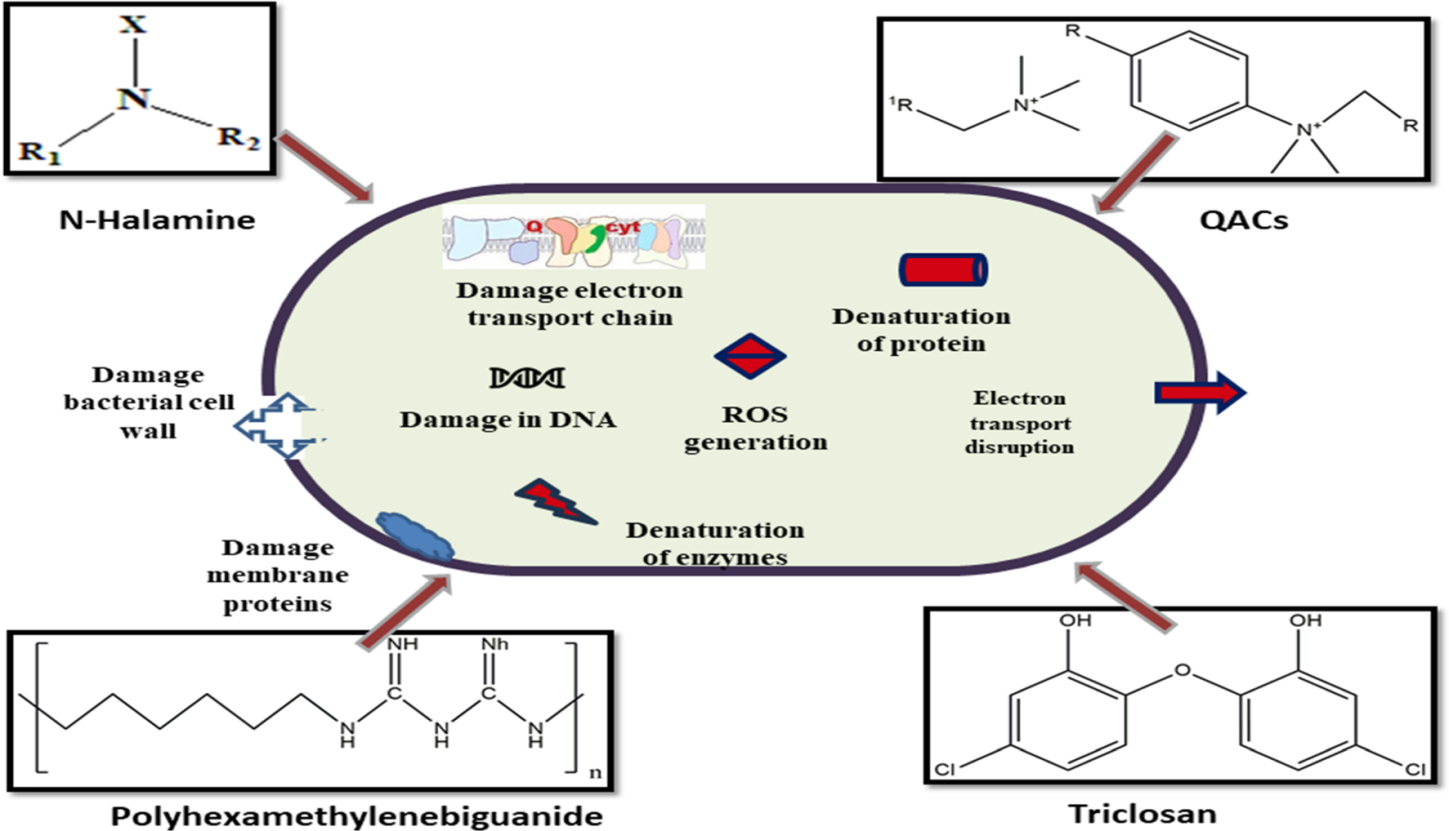
Mechanism of action of organic materials as antimicrobial agents for textiles. QACs inactivate bacteria or yeast by interacting with their cell membranes. PHMB damages membrane proteins. *N*-Halamines disrupt electron transport functioning. Triclosan damages intracellular proteins and stops lipid synthesis.

Triclosan is an aromatic compound containing aromatic rings attached with ethers, phenols, and chlorine in its structure, and it corresponds to halogenated phenoxy phenols.^70^ Due to its corrosive impact on bacterial enzymes involved in cell wall formation, triclosan demonstrates a broad spectrum of antibacterial and antifungal activity even at low doses. Cotton textiles were chemically engineered by Iyigundogdu along with other fellow scientists using 0.03% triclosan. According to their findings, 94% coronavirus reduction was seen after 2 hours of exposure.^47^ Triclosan is effective as antibacterial agent against variety of bacteria, the bacteriocidal activity of textiles is retained even after several washings. It is also an antiviral agent against influenza virus and several other viral strains Fig. 3.

Triclosan works by interacting with the bacterial cell membrane which causes the membrane to become porous so that the bactericidal triclosan can penetrate the cell. It binds to membrane and cytoplasmic targets and shows bactericidal action by disrupting the structure of bacteria. It causes bacteriostatic action by inhibiting the synthesis of fatty acids which are needed for the synthesis of cell membranes.^71^ NAD is required the synthesis of bacterial fatty acids, when triclosan binds to NAD to make a complex the fatty acid formation will not be facilitated and so bacteriostatic action occurs.

#### 5.2.2. Polyhexamethylene biguanide

Polyhexamethylene biguanide is known as PHMB, also is a common biocide with many uses. It has been employed as an antiseptic agent in hospitals to prevent wound infections, as a disinfectant in food production, and in swimming pool sanitation.^49^

The biocide interacts with the bacteria cells' surfaces when the fabric that has been treated with PHMB comes into touch with a bacterium and is then transmitted to the cytoplasm and cytoplasmic phospholipids in the bacterial membrane. Because this biocide is positively charged, it mostly reacts with negatively charged species, which causes aggregation and increases fluidity and permeability. Due to the interior material leaking out through the outer membrane, thus causes the death of the attacking pathogen Fig. 3.

PHMB can be applied during a paddy-cure process or immediately exhausted onto cellulose material. The interaction of the cationic molecule with anionic phospholipids within the bacterial cell wall, which results in cell wall destruction, is the basis for the antibacterial effect of positively charged compounds. Market-ready wound dressings currently include PHMB as an antibacterial ingredient.

#### 5.2.3. Quaternary ammonium compounds (QAC)

Worldwide, microbial infections affect people. Numerous quaternary ammonium compounds have been created, and in addition to being antibacterial, they are also antiviral, antifungal, and anti-matrix metalloproteinase Fig. 3. QACs are widely used as antiseptics, disinfectants, preservatives, and sterilization agents in a variety of settings, such as homes, medical facilities, and water treatment facilities.^72^ Because of differences brought about by the QAC's nature, the biocidal activity of QACs varies. The R group's characteristics, the presence of aromatic groups, the branching of the C chain, and the number of N atoms all play a role in the nature of QAC's distinctions.

Pathogens are known to be rendered inactive by quaternary ammonium salts, which are frequently employed to disinfect surfaces. The breakdown of the viral lipid membrane is assumed to be their mode of action against enveloped viruses. Based on shell disorder research, there is considerable worry that SARS-CoV-2 could be more stable in the environment. Combinations of antiviral agents, such as quaternary ammonium salts and phenolics, may be more successful in combating this virus.

QACs are membrane-active substances that interact with yeast's plasma membrane and bacteria's cytoplasmic membrane. They are also efficient against viruses that contain lipids due to their hydrophobic properties. Additionally, QACs engage in intracellular interactions and bind to DNA. Depending on the product composition, they are also effective against viruses and spores that don't include lipids. For lipophilic enveloped viruses, the virucidal mechanism of QACs appears to include breakdown or detachment of the viral envelope, followed by the release of the nucleocapsid. The greater affinity of encapsulated viruses for QAC through hydrophobic interactions may be the cause of viral envelope disruption. The antiviral activities of QACs against enveloped viruses have gained widespread recognition.^73^

One of the most promising methods to create antimicrobial biomaterials is to include quaternary ammonium moieties into polymers. Long chain alkyl substituent-containing silane-anchored QAC coatings have been widely used to porous surfaces to prevent pathogenic infections and manage bacterial metabolite-induced smells. The goal of these initiatives is to eradicate bacterial species efficiently before they have a chance to form a microbial biofilm and perhaps shield people from dangerous microbes. Sports goods, medical drapes and textiles, everyday apparel, and protective gear for odor and infection control are a few items that are treated with these substances.^74^ Si-QAC (silane quaternary ammonium compounds), according to some research studies show less or no harmful impact as compared to the single QACs that are more harmful. So QACs derived from silane polymers are more preferred and are commercially used as well.^75^

A few scientists at the National Centre of Biological Sciences, Bangalore, have developed a textile covering made of QACs that, so far, appears to be producing positive test findings. There are two ways to use the coating, one way after applying the solution to the fabric, such as a mask or PPE kit, heat fixing is used to assist molecules adhere to the fabric's surface. Another is fabrication of gloves, masks, PPE kits, *etc.*, can be done with textiles that have had the compound previously applied to them. Since there hasn't been any research to evaluate how it affects skin, it is now obvious that it cannot be administered as an ointment and may have harmful effects that are not yet recognized. Their research demonstrates that the solution effectively maintains its biocidal capability even after 25 washing cycles. Although the mask serves as a physical barrier, it also renders the virus inactive after contact.

#### 5.2.4. *N*-Halamines

Considerable research has been done on *N*-halamine antibacterial polymers throughout the past years. The substance have demonstrated exceptional stability in both wet and dry storage conditions. They are far less caustic than sodium hypochlorite and work well against a variety of microbes. *N*-Halamines are further distinguished by their low toxicity, affordability, and other advantageous qualities. They are especially well-known for their effectiveness, human safety, and eco-friendliness.^76^*N*-Halamine has been shown to be a beneficial addition to wound dressings, providing antibacterial qualities *via* easy and affordable procedures. Antimicrobial activity is obtained by coating or impregnating conventional non-antimicrobial wound dressings with *N*-halamine compounds that contain oxidative chlorine. These dressings become antibacterial by a simple chlorination procedure using diluted sodium hypochlorite solution. Dressings coated with *N*-halamine efficiently deactivate large quantities of *Pseudomonas aeruginosa* and *Staphylococcus aureus* germs during short contact times. When kept in opaque packaging, they exhibit stability under fluorescent lights for up to two months, extending their shelf life. These dressings outperform commercially available silver alginate dressings in terms of quick bacterial inactivation against both Gram-positive (*S. aureus*) and Gram-negative (*P. aeruginosa*) bacteria in 15 to 60 minutes. By eradicating unwanted bacterial development, *N*-halamine wound dressings have the potential to improve healing and avoid infections. In comparison to wound dressing materials, they are easily applied, affordable, stable when maintained correctly, effective against pathogenic germs during limited contact durations, and have little skin sensitivity.^77^

### 5.3. Carbon quantum dots (CQDs)

Carbon quantum dots are one of the nanomaterials that are used to prepare antimicrobial textiles. Carbon quantum dots can be made from nanotubes of carbon, graphite or graphene. One important metric for assessing the photoluminescence efficiency of carbon dots is their quantum yield, which is the ratio of photons emitted to photons absorbed. This characteristic is essential for figuring out how well CDs work in a variety of applications, such as optoelectronics, bioimaging, and sensing. A high quantum yield is preferred because it signifies effective light emission, which is crucial for applications that rely on fluorescence. Several variables, including surface passivation, the kind of precursors utilized, and the synthetic techniques used, can affect the quantum yield of CDs.^78^ Carbon quantum dots were prepared and immobilized on cotton fabrics for UV protection properties along with antimicrobial properties. In this approach, the microbicide ability of the prepared CQDs with different concentrations was evaluated against three different pathogenic species by using the inhibition zone technique. The MICs of the prepared CQDs were also evaluated. The estimated data significantly revealed that against all the tested bacterial and fungal species, CQDs (100 mg mL^−1^) showed the highest antimicrobial effect with MIC% of 100% against all the tested species.^79^ Another study offers a cutting-edge method for using carbon dots to industrialize fluorescent cotton textiles that are safely antimicrobial. For the first time, an infrared-assisted method for creating carbon dots from carboxymethyl cellulose was studied. The cotton fabrics that have been synthesized by this method can be used without risk in the industrialization of military and medical textiles. The current work is far superior to traditional methods for producing functional and medical textiles using metallic-based composites and is safer because carbon dots are biocompatible.^80^ Another study developed an inventive method for improving cotton fibers with integrated antibacterial and self-cleaning capabilities. The potential of silver-carbon quantum dot nanoparticles to fight bacterial infections was demonstrated by the elucidation of their antimicrobial properties. These nanoparticles demonstrated antimicrobial effects against both Gram-positive and Gram-negative bacteria that were concentration-dependent, indicating their considerable potential for a variety of applications, especially in textiles and healthcare.^81^ Carbon quantum dots can also be derived from polysaccharides such as cellulose or starch that can be used in the textile industry for biomedical applications. These polysaccharide-derived carbon quantum dots exhibit cytotoxic properties enabling their use for antimicrobial textiles along with this approach these carbon dots also enhance soil fertilization when released in the environment.^82^ Conclusively the carbon dots that are derived from polysaccharides are more productive and less harmful as compared to the synthetic ones because they utilize a green synthesis approach safer for health and environment.

### 5.4. Disadvantages of synthetic antimicrobial agents used on textiles

After the COVID-19 pandemic, awareness about the use of antimicrobial textiles has increased which ultimately increased the demand for more safe and eco-friendly antimicrobial textiles. The pathogen protective textiles are not only used in medical wear but they are also used in everyday life, so these textiles must not be toxic to humans, should have a broad spectrum of antimicrobial potency, should not cause damage to the inherent fabric properties, should be able to withstand fabric manufacturing procedures, and its antimicrobial durability should be stable throughout the fabric's use life.^83^

**Fig. 4:**
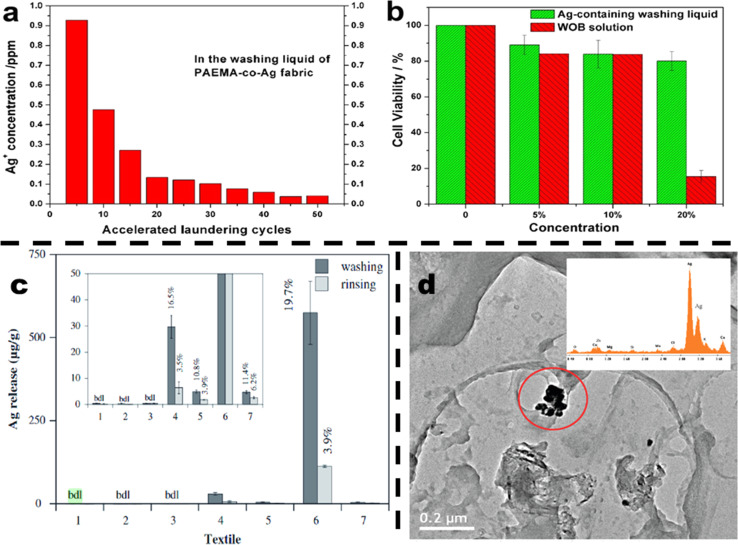
(a and b) Reproduced with permission from https://creativecommons.org/licenses/by-nc-sa/4.0/(a) Ag ions concentration measured by ICP-MS in washing liquids of the PAEMA-co-Ag fabric in accelerated laundering test; (b) the cytotoxicity of washing liquid on human HaCaT cells. The cells were exposed to different concentrations of PAEMA-co-Ag washing liquid at 5th. Accelerated laundering cycle and WOB detergent for 48 h ref. 84 copyright (2014) https://nature.com. (c) Reproduced with permission from https://creativecommons.org/licenses/by/4.0/quantity of silver released from the seven textiles at the time of washing as well as rinsing. The inset presents an expanded outlook of the lower concentration range ref. 85 copyright (2020) MDPI. (d)“TEM” image of colloidal material from sock wash water. Inset: EDX confirmation that the dark particles within the circle are predominantly silver. Reproduced with permission from “Reprinted with permission from {ref. 86}. Copyright {2008} American Chemical Society”.

Synthetic antimicrobial agents show excellent antibacterial and antiviral properties but there are also some adverse effects associated with these chemicals. Adverse effects are not only associated with human bodies but they also cause several environmental problems. Chemical agents from textiles can detach from the textiles during washing and may enter the water bodies thus causing death of aquatic life and may also cause deterioration of crops using contaminated water. Fig. 4 shows some results from the literature showing the release of harmful antimicrobials in water bodies during washing cycles that can pollute the environment and also cause health risks. Along with results of the leaching of antimicrobials some effects of chemical-based textiles are given in Table 2.

**Table 2:** Harmful effects of different synthetic antimicrobial agents used for textiles

Indications	Synthetic antimicrobial agent	Effects	Ref.
Irritation	Triclosan	Triclosan-embedded textiles can cause skin irritation	87
	Silver NPs	Eye irritation and allergic contact dermatitis	88
	Cu and Zn NPs	Release of Cu and Zn ions from textiles causes skin irritation and inflammation	89
Water toxicity	Triclosan	Triclosan may detach from the textile during washing and cause its bioaccumulation in fish	90
	QACs	QACs are toxic to aquatic life when released in water bodies	91
Bacterial resistance	Triclosan	Due to excessive use in textiles and health care products, micro-organisms are developing resistance against triclosan	75
	QACs	The presence of QACs in polluted environments develops resistance in bacteria	92
	Ag, Cu, Ti NPs	Bacteria develop resistance against metal NPs by developing special structures	93
Photochemical deterioration	Triclosan	When released in water bodies triclosan converts to 2,8-dichlorodibenzo-*p*-dioxin which is highly toxic	94

## 6. Natural antimicrobial agents for textiles

**Fig. 5:**
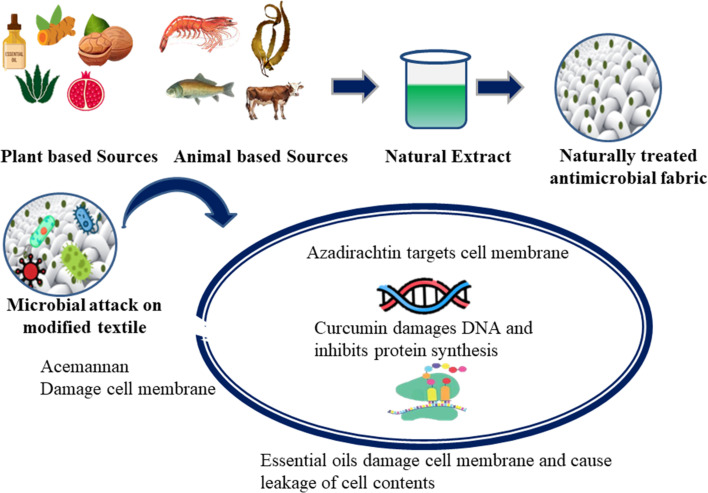
Mechanism of action of natural antimicrobial agents showing different natural antimicrobials target different cell functions.

There are some naturally occurring agents which are used in the textile industry due to their efficiency against a variety of viruses. The natural antiviral agents are safe, non-toxic and inexpensive as compared to the synthetic antiviral agents. The natural antiviral agents include animal extracts, plant extracts and some essential oils (Fig. 5). There are different mechanisms of action of the antiviral agents through which they either stop the growth of the virus or kill the virus. The different natural antiviral agents work through different mechanisms of action. The common mechanism of action include the inhibition of virus replication by inhibiting the synthesis of DNA and inhibition of attachment of the virus to the target cell.

In the past, antimicrobial finishing treatments for textiles included triclosan, quaternary ammonium compounds, and nanosilver. However, because they are synthetic, the majority of them are pricey and cause environmental issues. Natural textile finishes with additional value, especially for medical apparel, are highly valued because modern textile processes prefer eco-friendly chemicals for antimicrobial textile finishing. When treated with natural chemicals, cotton and other natural fibers have strong antibacterial properties. Although silk is thought to possess inherent antibacterial properties, a broad spectrum of microorganisms may not be affected. However, use of natural colors such as curcumin, or derived from plants such as *Terminalia catappa*, *Morinda citrifolia*, *Tectona grandis*, *Artocarpus heterophyllus*, *etc.*, has effectively induced a notable degree of antibacterial action against microbes.^95^ Green synthesis is used for producing antimicrobial textiles which has a safe potential towards disease protection without damaging health.

### 6.1. Plant extracts

#### 6.1.1. Acemannan from aloe vera

Anthraquinones are the active agents found in aloe vera which are responsible for antibacterial as well as antiviral activity.^108^ Aloe vera is a well known antibacterial and antifungal agent that has been used since very long times to treat infections. Aloe vera is referred to as a “healing plant” because of its advantages in the treatment of wounds, sun protection, anti-oxidant properties and anti-microbial properties.^109^ Aloe vera is frequently utilized to create various textile composites for the fields of tissue engineering, medical textiles, and wound healing. Aloe vera treated fabric show significant decline in microbial growth as compared to the untreated fabric. According to Jothi bacterial growth reduction is directly proportional to the increase in concentration of aloe vera solution applied to the fabric. Aloe vera gel solutions with different solution concentrations, 1, 2, 3, 4 and 5 g l^−1^, were applied to the cloth. Aloe vera treatment applied to fabric at a rate of 5 g per liter resulted in strong anti-microbial activity against *S. aureus*.^96^ The active ingredients in the aloe vera gel function as a powerful bactericidal agent on the fabric, preventing the growth of the Gram-positive bacteria. The active antibacterial component in aloe vera is acemannan which is a long chain polymer which shows antibacterial as well as antiviral properties Table 3.

**Table 3:** Different natural antimicrobials used for textile applications

Antimicrobial agents	Textile material	Application	Active against	Ref.
**Plants**				
Acemannan	Wound dressings, cotton and silk fabrics	Corona virus, *S. aureus*, *E. col*, *Influenza virus*	Virus and bacteria	96 and 97
Azadirachtin	Cotton fabrics	*S.aureus*, *E. coli*, *Pseudomonas aeruginosa*, *Candida albicans*	Bacteria	98
Curcumin	Fibers	*S.aureus*, *S.sonnei*, *E. coli*	Bacteria	99
Punicalagin	PPE	*S.aureus*, *Klebsiella pneumoniae*	Bacteria	100
Methanolic extracts from walnut	Cotton fabrics	*S. aureus*, *E. coli*	Bacteria	101
Essential oils	Medical and sports wear	Corona virus, *S. aureus*, *E. coli*, *S. sonnei*, *Klebsiella pneumoniae*	Bacteria and virus	102

**Animal extracts**				
Chitosan	Cotton and wool fabrics	Gram-positive and gram-negative bacteria and fungi	Fungi and bacteria	103 and 104
Alginate	Surgical masks, wound dressings and gloves	Gram-positive and negative bacteria, Herpes simplex virus	Bacteria and virus	105 and 106
Collagen hydrolysate	Cotton	*Staphylococcus aureus* and *Escherichia coli* and antifungal activity against *Candida albicans*	Bacteria and fungi	107

Aloe vera also shows excellent healing properties which make it suitable to be used as wound dressings. Mannose, an ingredient in aloe vera, promotes faster wound healing and enhanced macrophage activity. Rapid fibroblast proliferation is produced by macrophages, which accelerates tissue growth and reduces the healing time.^110^

#### 6.1.2. Azadirachtin from neem

Azadirachtin is the active antibacterial agent found in neem, it is a complex tetranortriterpenoid limonoid which causes bactericidal action due to cell wall breakdown.^111^ Neem is an evergreen plant native to India and has amazing medicinal, antibacterial, and insect-repelling qualities. As a result, it has been utilized as a traditional medicine since ancient times to treat a variety of human diseases. The study's findings indicate that fabrics treated with plant combinations; *Terminalia chebula* (Myrobalan) along with *Aloe barbadensis* (Aloe vera) and *Azadirachta indica* (Neem) had bacterial reduction values that ranged from 59% to 69% against Gram-negative bacteria and from 58% to 64% against Gram-positive bacteria.^112^ Since neem extracts have both pest-repelling and the potential to support bacterial growth, they are frequently utilized in the production of antimicrobial textiles. Numerous bacteria, including *Staphylococcus aureus*, *Escherichia coli*, *Pseudomonas aeruginosa*, and *Candida albicans*, have been demonstrated to be inhibited by neem extract-treated textiles. Textiles treated with neem extract retain their antibacterial properties for a number of washings.

#### 6.1.3. Curcumin from turmeric

Curcumin is the bioactive agent that is extracted from turmeric that is used for synthesis of antimicrobial textiles.^113^ Turmeric also known as (*curcuma longa*) is an antimicrobial plant that has been used for ages as a food colourant and to dye fabrics due to the presence of a pigment called curcumin. It is also known as food coloring agent. Turmeric shows antibacterial activity due to the presence of active phenolic and hydroxyl groups.^114^ It has been demonstrated that curcumin interacts with peptidoglycan to break down the cell membrane and cause cell lysis. Curcumin may also interact with the cell wall to compromise its integrity and provide bacteriostatic effects. A study in 2005 showed antibacterial activity of turmeric in dyed wool fabrics. Antibacterial properties of treated fibers against *S. aureus*, *S. sonnei*, *E. coli* were observed.^115^ Another study conducted in 2010 employed the pad-dry cure method to apply microcapsules containing turmeric plant extract to silk and cotton fabrics. Results revealed that fabrics treated with turmeric extract have antibacterial properties against all types of bacteria that had been researched.^99^ Another study conducted in 2010 used silver nanoparticles along with turmeric extract and immobilized the ions on cotton fabric. The results showed enhanced antimicrobial activity as compared to the bare silver nanoparticles. This procedure makes the silver nanoparticles more eco-friendly.^116^

#### 6.1.4. Punicalagin from pomegranate

Punicalagin is the bioactive agent found in pomegranate peels responsible for antimicrobial activity.^117^ Pomegranate shows antimicrobial activity when applied to textiles. It has been used for a very long time to dye fabrics. The antimicrobial activity is also due to the presence of active compounds such as tannins and ellagic acid. A study conducted in 2009 used pomegranate-dyed fabric to test the bactericidal activity, the results showed that the test bacteria in solution were inhibited by using a natural extract combination *P. granatum* (pomegranate, *A. cepa* (onion) and *R. tinctorum* (rose madder)). On a sample of wool coloured with pomegranate, there is a 4–80% reduction in bacterial growth.^118^ Another study tested the antibacterial efficacy of cotton, wool, and silk fibers dyed with pomegranate extract against *Staphylococcus aureus* and *Klebsiella pneumonia* bacteria for 60 minutes at 80 °C. The effectiveness of pomegranate extract against *Staphylococcus aureus* is 99.9–96.8%, while it is 95.7–99.9% effective against *Klebsiella pneumonia*. Pomegranate-dyed cotton fibers had bacteriostatic decrease rates against *Staphylococcus aureus* of 99.9% and *Klebsiella pneumonia* of 95.8%.^100^

#### 6.1.5. Methanolic extracts from walnut

The walnut plant is also used as a colouring agent to dye clothes since ancient times. Walnut shows bactericidal activity due to the presence of methanolic extracts, phenols and flavonoids, these compounds are also responsible for anti-inflammatory and anti-oxidant activity. Its bacterial activity was studied in 2013 where walnut shells were utilized to naturally color cotton while chitosan was present. Results revealed that the fibers had been given an antimicrobial treatment.^101^ In another study wool fibers were given an antimicrobial finish by creating *in situ* Ag/Cu_2_O/ZnO nanoparticles on their surface, which were then dyed with two natural dyes derived from pomegranate peel and walnut green husk. The natural colors that were isolated showed antibacterial properties as well. The samples treated only with punicalagin from pomegranate peels and methanolic contents from walnut green husks had antibacterial activity of 65% and 35%, respectively. After being dyed, treated wool yarns with inorganic salts showed excellent (nearly 100%) antibacterial activity. Additionally, the treated samples' wash and light fastness qualities ranged from very good to exceptional^119^Table 3.

#### 6.1.6. Essential oils

Essential oils are made from the extract of different parts of plants such as roots, bard and leaves. These are concentrated solutions of aromatic components in the plants. They can be taken from plants with flowers like jasmine, rose, and lavender as well as leaves like eucalyptus and thyme. The process by which textiles are incorporated with essential oils is also known as aromatherapy. Essential oils are mostly combinations of terpenes, sesquiterpenes, oxygenated derivatives, aldehydes, oxides, phenols, ethers, acids, and ketones are extremely complicated and are responsible for imparting fragrance.^120^ The essential oils are efficient against a variety of diseases due to the presence of various aldehydes, phenolics, terpenes, and other antibacterial components. Terpenoids and hydrocarbon terpenes are the primary active ingredients in essential oils. Because of their antibacterial, antimalarial, antineoplastic, and other pharmacological qualities to treat human ailments, they have been frequently utilized in conventional herbal remedies. They have found usage as disinfectants for medical equipment and surfaces or are used to prevent nosocomial infection due to their bactericidal and fungicidal effects.^121^ Essential oils are difficult to handle which restricts their use in the textile sector, however, microencapsulation is a technology which uses capsulated essential oils for impregnation on textiles thus controlling their release rate which extends their antimicrobial effect. Various essential oils are used for antimicrobial textiles some compounds along with their encapsulated materials are listed in the Table 4.

**Table 4:** Essential oils used for antimicrobial textiles

Essential oil/core material	Capsule material/shell material	Antimicrobial effect	Ref.
Pomegranate rind	Chitosan/Gum Arabic	Medical textiles	122
Jojoba oil	Ethylcellulose	Knits for burnt skin	123
Clove thyme and cinnamon	Alginate	Medical textiles	124
Cologne essential oil	Methyl methacrylate polymer	Cotton fabrics	125
Peppermint oil	Alginate	Cotton fabrics	126
Moxa oil	Gelatin Arabic gum	Cotton fabrics	127

### 6.2. Animal extracts

#### 6.2.1. Chitosan

Chitin is a polymer obtained from micro-organisms, sea animals and insects. Chitosan is an eco-friendly polymer obtained from the diacylation of chitin and is widely used in the textile industry in finishing processes. Chitosan is incorporated into the fabric and the fabric becomes antimicrobial. The incorporated chitosan works as an antimicrobial by two approaches. Firstly, the chitosan inhibits the entry of the virus by lowering the surface energy. Secondly, the virus can be restricted to the textile surface by destroying the DNA. The chitosan destroys the DNA by entering in the virus through chitosan oxidation and dissolving the lipid structure of viral cells. The cationic nature of chitosan shows antibacterial action involving attachment to the negatively charged bacterial cell wall and disrupting the cell, changing the permeability of the membrane. This is followed by attachment to DNA, which inhibits DNA replication and ultimately results in cell death.^128^ Chitosan can be applied to the fabric by using different application techniques such as layer by layer technique, coating, impregnation, press-rolling process, wet spinning. ChSN/carboxymethylcellulose composite is a type of chitosan which is applied on the fabric by using layer by layer technique and shows antiviral activity against coronavirus with an efficiency of 99.99%.^129^

#### 6.2.2. Alginate

Alginate present in brown seaweed as a bioactive compound is used in the textile industry for medicinal products. Alginate is not typically used in the textile rather it is used in the broader strategy to create antiviral textiles.^130^ The hybrid of alginate copper sulphate coatings on the different types of textiles such as fibers, yarn and fabrics are used to deactivate the coronavirus. The purpose of alginate is to enhance the biocompatibility. The alginate also adjusts the availability of the metal ion. This coating proves to be effective against coronavirus if it is applied in multilayers and its efficiency against coronavirus is 99.99%. Alginate fibers are used in wound dressings as they have the ability to absorb liquid in the wound which promotes healing also sodium and calcium exchangeability at wound surface makes alginate suitable to be used on wounds.^131^

#### 6.2.3. Collagen hydrolysate

Collagen is a fiber found in bones and tissues. It has antibacterial properties which make it suitable to be used in medical textiles for coating fabrics, wound dressings, hydrogel dressings and implantable medical devices. Hydrolyzed collagen derived from bovine leather by-products was loaded with ginger essential oil and electrospun to produce bioactive nanofibers. Antibacterial activity against *Staphylococcus aureus* and *Escherichia coli* was also evaluated, as was antifungal activity against *Candida albicans*. Data demonstrate that hydrolyzed collagen nanofibers infused with ginger essential oil can be employed in medical, pharmaceutical, cosmetic, and specialized applications.^132^ Collagen hydrolysates from rabbit skins and cow tendons were combined with chitosan *via* the coaxial electrospinning method in order to be used as possible wound dressings. The electrospun bioactive composites' antibacterial activity demonstrated the effectiveness of the chitosan-based bovine collagen hydrolysate-based nanofibers against various bacterial species.^133^

## 7. Conclusion

Chemically modified functional textiles are a good addition to controlling infections and various pathogens that cause diseases. A large number of synthetic and natural chemicals are being used with proven efficiency in controlling germs. Textiles treated with synthetic chemicals when washed or discarded after use can emit chemicals, thus posing harmful effects to the environment. Natural products play a pivotal role in developing a sustainable and environment-friendly approach to meet the sustainable development goals. Together, these plant extracts provide a sustainable and effective approach to the production of antimicrobial fabrics, promoting medical textiles, wound care, and protective fabrics. Their natural background and multi-layered benefits make their continued quest for innovative, eco-friendly solutions in the textile industry invaluable. Animal polymers such as chitosan, alginate and collagen hydrogen, have shown considerable potential in the textile industry, especially in the preparation of antimicrobial and antiviral fabrics. A versatile approach to blocking viral access, destroying viral DNA, and disrupting the walls of bacterial cells. It is very effective in the production of antiviral textiles. These animal extracts contribute to advancing the development of textiles that can play a critical role in healthcare and protective applications. Research must be directed towards investigating the effect of natural antimicrobials on microbes, their antimicrobial efficiency, their safe disposal and shelf life. Future investigations required to explore ways for the disposal of chemically modified textiles to prevent the leaching of chemicals into the environment.

## Author contributions

Conceptualization: Muhammad Zubair,: writing-original draft: Muhammad 650 Zubair; Gulafza; data curation; Sajjad Hussain Sumrra, Muhammad Asif Hanif, visualization: Aqsa BiBi, 651 Zoya Afzal; review & editing: Aqsa BiBi Zoya Afzal, Mujahid Farid, Bedigama Kankanamge Kolita 652 Kama Jinadasa.

## Conflicts of interest

The authors declare no conflict of interest.
